# Development of New Formula Microcapsules from Nutmeg Essential Oil Using Sucrose Esters and Magnesium Aluminometasilicate

**DOI:** 10.3390/pharmaceutics12070628

**Published:** 2020-07-04

**Authors:** Inga Matulyte, Giedre Kasparaviciene, Jurga Bernatoniene

**Affiliations:** 1 Department of Drug Technology and Social Pharmacy, Lithuanian University of Health Sciences, LT-50161 Kaunas, Lithuania; inga.matulyte@lsmuni.lt (I.M.); giedre.kasparaviciene@lsmuni.lt (G.K.); 2 Institute of Pharmaceutical Technologies, Medical Academy, Lithuanian University of Health Sciences, LT-50161 Kaunas, Lithuania

**Keywords:** nutmeg essential oil, sodium alginate, magnesium aluminometasilicate, sucrose esters, microcapsule, encapsulation, encapsulation efficiency, extrusion, polysorbate 80

## Abstract

Essential oils are volatile liquids which evaporate and lose their pharmacological effect when exposed to the environment. The aim of this study is to protect nutmeg essential oil from environmental factors by encapsulation (shell material, sodium alginate) and determine the influence of crosslinker concentration (2%, 5% calcium chloride), different emulsifiers (polysorbate 80, sucrose esters), and magnesium aluminometasilicate on microcapsule physical parameters, encapsulation efficiency (EE), swelling index (SI), and other parameters. Nutmeg essential oil (NEO)-loaded calcium alginate microcapsules were prepared by extrusion. The swelling test was performed with and without enzymes in simulated gastric, intestinal, and gastrointestinal media. This study shows that the crosslinker concentration has a significant influence on EE, with 2% calcium chloride solution being more effective than 5%, and capsules being softer with 2% crosslinker solution. Using sucrose esters, EE is higher when polysorbate 80 is used. The swelling index is nearly three times higher in an intestinal medium without enzymes than in the medium with pancreatin. Microcapsule physical parameters depend on the excipients: the hardest capsules were obtained with the biggest amount of sodium alginate; the largest with magnesium aluminometasilicate. Sucrose esters and magnesium aluminometasilicate are new materials used in extrusion.

## 1. Introduction

Essential oils are unstable materials; they have to be protected from environmental influence because when volatile compounds evaporate spreading in the air, the effect of the essential oil is reduced, and its use is pointless. Essential oils contain many chemical compounds (monoterpene hydrocarbons, oxygenated monoterpenes, sesquiterpene hydrocarbons, oxygenated sesquiterpenes, and others) which are not stable [1,2,3]. Essential oils not only have an odor or flavor [4] but they also have antibacterial [5,6], antioxidant [2,7], and many pharmaceutical effects: they decrease pain [8,9], reduce inflammation [10], and promote wound healing [11,12]. For instance, for nutmeg, its essential oil has antibacterial, antioxidant, anti-inflammatory, and insecticidal effects [1,13]. It is important to protect the properties of the essential oil in order to not lose its biological effect. Therefore, in this research, two new agents were used—magnesium aluminometasilicate (absorbent) and sucrose esters (emulsifier).

The physical parameters of essential oil microcapsules are important for their quality. The diameter of wet or dry microcapsules is one of the most important parameters that determines whether a properly sized microcapsule is formed (nano- or micro-capsules can be formed) [14]. The most important parameter is the encapsulation efficiency [15,16,17]; it shows how much of an essential oil is encapsulated and how it is protected from environmental factors. When examining the influence of excipients on microcapsules, it is important to determine the firmness and swelling of the capsules [17,18].

In this research we chose two emulsifiers: polysorbate 80 (Tween 80) and sucrose esters (Sisterna SP70). Both emulsifiers are non-ionic surfactants that have a high HLB value (HLB = 15), and are used for emulsification of oil in water [19]. Polysorbate 80 is often used as an emulsifier, it has high ability to emulsify and is well known, but Sisterna SP70 has never been used as an excipient for microencapsulation of essential oil [15,20,21]. It will be a new material in experiments with nutmeg essential oil (NEO)-loaded calcium alginate microcapsules, manufactured by using an extrusion method. Sucrose esters are white powders, soluble in water and frequently used in food or cosmetic industry [22,23,24,25,26], so it can be predicted that sucrose esters would work well in other areas, such as pharmaceuticals, when formulating drugs or nutritional supplements with essential oils or emulsions. Applying theoretical knowledge of this material, a new technology of nutmeg essential oil microencapsulation can be developed.

Microencapsulation is an effective method to protect unstable materials. The core of active substance is protected by excipients which formulate a shell [27], so the essential oil is safe from environmental factors (temperature, moisture, oxygen exposure) and retains active compounds [28]. There are many methods of microencapsulation and they are divided into two main groups: chemical and physical. The chemical microencapsulation methods include polymerization, suspension, in-situ emulsion, and dispersion, while physical methods include spray drying, coacervation, pan coating, air suspension, extrusion, lyophilization, and others [4,27,29,30]. Some methods, such as pan coating and air suspension, are limited to encapsulating liquid substances. Accordingly, essential oil as a core material reduces the choices of methods because liquids have different a structure compared to solid substances, and the method of microencapsulation must be selected for specific properties [30]. In terms of the physical method extrusion, it is a method which is introduced as an action of pushing out [31]. Extrusion can be easily modified: a medical syringe with a needle can be used for the formation of microcapsules [32] or a specific device, like a syringe pump. [4,33,34]. Certainly using a syringe pump or other similar devices for extrusion facilitates the work and makes it easier to maintain a level playing field throughout the experiment [35].

Particle size of microcapsules prepared using extrusion method ranges from 250 µm to 2500 µm. Of course, the size also depends on devices and excipients used. In practice, microcapsules can even be 2–3 mm in diameter. The average diameter of microcapsules using alginate and containing *Lactobacillus reuteri* was 2.37 mm [31]. The Ca-alginate beads (palm oil encapsulation), when using extrusion, were 1830 µm in diameter [36]. The diameter depends on the quantity of shell material, which will define the size of the capsule, as well as the technique that is used and also the number of other excipients (if they are added).

The choice of shell materials is large, and there are many polymers which could coat an active ingredient. For example, a natural polymer gelatin was used to prepare rosemary and thyme essential oil microcapsules by coacervation [4]. Gelatin coated the essential oil drops and prevented the loss of active compounds. Other shell materials could be collagen, chitosan, sodium alginate, and other substances that can form a shell [28,29,37,38]. In terms of sodium alginate as a shell material, it is a biopolymer with unique properties. Sodium alginate is a polysaccharide that is chemically stable, can form strong gel barriers from water, is pH sensitive, and is functional not only in pharmaceutical industry but also in food industry as an additive, which acts as a thickener and a gelling agent [17,28]. Sodium alginate from brown algae is not the only agent of alginate used in industry, magnesium alginate or calcium alginate can be used as well. Calcium alginate is insoluble in water and usually is used as a co-gelling agent; a small amount of this material can make the sodium alginate gel solid and thick because calcium (Ca^2+^) ions exchange sodium (Na^+^) ions, alginate chains connect, and a thick gel forms. Calcium chloride solution also works; it is one of the crosslinkers and forms a microcapsule shell [17,31,38,39,40].

Surface active materials such as stabilizers and emulsifying agents are added to essential oil emulsions, which do not allow the emulsions to layer out and separate into phases [41]. Sodium alginate has a poor emulsifying capacity [17] and to prepare a stable emulsion an emulsifier has to be used. Yet research has not revealed whether the unstable emulsion affects the amount of encapsulated essential oil. 

The aim of this study was to prepare microcapsules with nutmeg essential oil by extrusion using sodium alginate as a shell material and two non-ionic surfactants (polysorbate 80 and sucrose esters) as excipients which form and stabilize the emulsion, to evaluate the physical parameters of microcapsules and compare the influence of two emulsifiers on capsules’ parameters, and, finally, to calculate the essential oil encapsulation efficiency. We chose to determine the influence of magnesium aluminometasilicate on encapsulation efficiency by using it as an absorbent because in previous studies it has been found to absorb volatile compounds and increase the yield of essential oils [42]. In addition, it is important to compare the swelling of microcapsules with different excipients in different media, and to determine the impact of emulsion stability on the encapsulation efficiency of the nutmeg essential oil.

## 2. Materials and Methods 

### 2.1. Materials

The nutmeg essential oil (Sigma-Aldrich, Singapore) from *Myristica fragrans* seeds was used as the core material in microcapsules. The essential oil is a limpid, light yellowish liquid and has a specific odor. Polysorbate 80 (Tween^®^ 80, Roth, Germany) and sucrose esters (Sisterna^®^ SP70, Sisterna, The Netherlands) were used as surfactants which stabilized the nutmeg essential oil emulsion. As the shell material, alginic acid sodium salt from brown algae (Sigma-Aldrich, Shanghai, China) was used. Calcium chloride (Farmalabor, Italy) salt was used to formulate microcapsules as a crosslinker which linked sodium alginate chains and formed a solid gel. Magnesium aluminometasilicate (Neusilin^®^ US2, Fuji Chemical Industries Co., Osaka, Japan) was used as an absorbent of volatile compounds. Ethanol 96% (Vilniaus degtinė, Vilnius, Lithuania) was used as a solvent for encapsulation efficiency determination. Distilled water was used throughout the experiment. Potassium dihydrogen phosphate (Supelco, Darmstadt, Germany), sodium hydroxide (Sigma-Aldrich, Munich, Germany), pancreas powder (Mezym 20000 U, Berlin-Chemie, Berlin, Germany), sodium chloride (Sigma-Aldrich, Munich, Germany), pepsin powder (Sigma-Aldrich, Soborg, Denmark), and hydrochloric acid (36% (Standard, Krakow, Poland) were used to produce simulated intestinal and gastric juices. 

### 2.2. Nutmeg Essential Oil Emulsion Preparation0

First, 4% sodium alginate solution was prepared from distilled water and alginic acid sodium salt. It was used throughout the experiment for emulsion preparation as the shell material. Emulsion with polysorbate 80 was prepared as follows: 4% sodium alginate solution (5 g, 10 g, and 15 g) was diluted with distilled water (10 mL, 5 mL, 0 mL), a surfactant (0.5 g, 1 g, 1.5 g) was added, everything was stirred with a magnetic stirrer MSH-20A (Witeg, Germany) for 10 min and then the nutmeg essential oil (0.5 g, 1 g, 1.5 g) was added. The emulsion was stirred for 15 min. The absorbent magnesium aluminometasilicate (0.2 g and 0.4 g) was used in several samples; this material was added before the essential oil.

**Figure 1:**
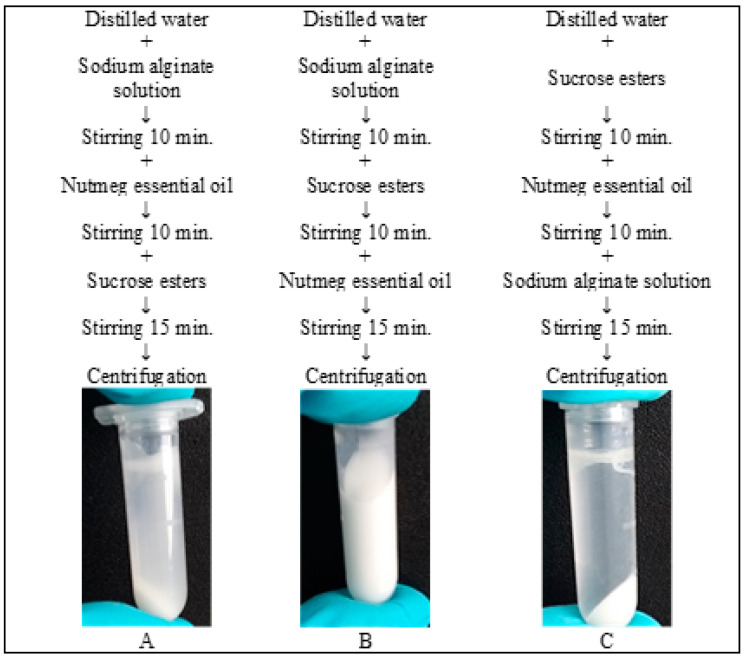
Three methods to produce emulsion with sucrose esters.

The emulsion with Sisterna SP70 was prepared by three different methods (Figure 1). 

The stability of each emulsion was tested using a centrifuge Sigma 3-18KS (Sigma Laborzentrifugen GmbH, Osterode am Harz, Germany). The test was carried out in these conditions: 3000 rpm, temperature 23 °C, and duration 5 min. All samples were tested three times.

### 2.3. Nutmeg Essential Oil Emulsion Stability

The emulsion with nutmeg essential oil was prepared using two different emulsifiers and one extra excipient, magnesium aluminometasilicate, excluding the main sheathing material sodium alginate. To determine the emulsion stability, the centrifuge test was performed and the quantity (%) of non-layered emulsion (centrifugation index (CI)) was calculated by the equation:

(1) $$CI\;\left(\%\right)=\frac{V_{e}}{V_{i}}\times 100$$

where *V_e_* is the volume of the remaining emulsion after centrifugation and *V_i_* is the volume of the initial emulsion. The test was repeated three times and the CI average was calculated. Test parameters: 3000 rpm, temperature 23 °C, and duration 5 min.

### 2.4. Emulsion and Microcapsule Morphology Study

Morphology of the emulsion and microcapsules was examined under light microscope Nikon H550S (Nikon, Chiyoda-ku, Japan) with incorporated monitor display. The emulsion and dried microcapsules were put (emulsion was dropped) on a microscope slide and observed with objective magnification of 4× and 10×. Using NIS-Elements D3.2 program, the image was captured, and it showed the essential oil drop’s general view, size, and microcapsules sphere.

The homogenizer IKA^®^ T18 digital Ultra Turrax^®^ (IKA^®^-Werke GmbH & Co. KG, Staufen, Germany) was used to compare emulsion’s morphology before and after homogenization.

### 2.5. Encapsulation of Nutmeg Essential Oil by Extrusion

Microcapsules were prepared by extrusion method. The medical syringe (Jiangsu Zhengkang Medical Apparatus, Shanghai-Nanjing Railway, China) was used as an ejector to prepare droplets for microcapsules. The drops of emulsion were ejected from the needle into the crosslinker solution. The height from the needle to the solution surface was 10–15 cm. The crosslinker solution was made of two concentrations: 2% and 5% of calcium chloride. Microcapsules were prepared using a magnetic stirrer. NEO-loaded calcium alginate capsules in the crosslinker solution were stirred for 15 min for curing, then the microcapsules were filtered using filter paper and washed with distilled water. Manufactured capsules were left to dry at room temperature (20 ± 2 °C) for 24 h. Dried microcapsules were stored in sealed tubes until further tests.

### 2.6. Microcapsule Physical Parameters: Firmness and Size

Using texture analyzer TA.XT.plus (Texture Technologies, Brewster, NY, USA), the force of firmness was measured on freshly made NEO-loaded microcapsules. P/100 platen was used as a probe. The force required to compress a 2 mm microcapsule (g force) was measured. The maximum force of the device was 6500 g force. For one sample, 10 units of microcapsules were used. Three measurements were taken, and the mean and standard deviation calculated.

Microcapsules’ size was measured using Digital Caliper micrometer (BGS technic, Wermelskirchen, Germany). The diameter was determined in dried and freshly made capsules. Then, 30 units of capsules were measured, and the mean and standard deviation calculated.

### 2.7. Preparation of Nutmeg Essential Oil Standard Graph

The standard graph of calibration was prepared by UV Spectrophotometer UV-1800 (Shimadzu, Kyoto, Japan). Standard solutions were made from the nutmeg essential oil which was diluted with 96% ethanol. The solution concentrations were from 0.4 mg/mL to 2.4 mg/mL. The absorbance was measured at 274 nm [43] and the essential oil encapsulation efficiency in microcapsules determined. 

### 2.8. Determination of Encapsulation Efficiency

The dried NEO-loaded microcapsules (0.1 ± 0.05 g) were dissolved in 96% ethanol (5 mL). Sodium alginate capsules are not soluble in ethanol, but the essential oil is. After, the 0.2 mL of solution was poured in a cuvette and 3 mL of 96% ethanol were added. The absorbance was measured at 274 nm. The essential oil dissolution from microcapsules was measured after 1 h and 24 h. 

Encapsulation efficiency was calculated as follows: the quantity of essential oil in microcapsules q*_p_* (mg/mL), determined using a spectrophotometer, divided by the quantity of total essential oil added to microcapsules q*_t_* (mg/mL), and multiplied by 100. The encapsulation efficiency (EE) was determined by the formula:

EE (%) = q*_p_*/q*_t_* × 100 (2)

### 2.9. Swelling Characteristic of Nutmeg Essential Oil Microcapsules

Swelling of NEO-loaded microcapsules was performed in gastric, intestinal, and gastric-intestinal media. The composition of simulated gastric juice (SGF) contained 2.0 g of NaCl, 80 mL of 1M HCl solution, 3.2 g of pepsin, and distilled water up to 1000 mL (pH = 1.2); simulated intestinal juice (SIF) contained 6.8 g of KH_2_PO_4_, 77.0 mL of 0.2M NaOH solution, 10 g of pancreas powder, and distilled water up to 1000 mL. The mixed medium was made as follows: NEO-loaded microcapsules were left to swell in SGF medium for 1 h, then the capsules were transferred into the SIF medium. The NEO-loaded microcapsules were weighed at successive time intervals of 0, 15, 30, 60, 90, 120, 180, 240, 300, and 1440 min when using SGF and SIF media separately, and at the intervals of 0, 15, 30, 60, 75, 90, 120, 180, 300, and 1440 min when the medias were used together. The swollen microcapsules were removed and filtered using a metal mesh and dried with a paper towel to eliminate the excess fluid. To determine the swelling index (SI), the following formula was used:

(3) $$Sweling\;index\;\left(\%\right)=\frac{W_{s}-W_{i}}{W_{i}}\times 100$$

where *W_s_* is the weight of swollen NEO-loaded microcapsules at the time, *W_i_* is the dried microcapsules’ weight. The swelling process was carried out three times.

### 2.10. Statistical Analysis

Data are presented as the mean ± SD (except the SD of swelling). Statistical analysis was performed using Student’s *t*-test. The results were significant when *p* < 0.05. 

## 3. Results and Discussion

### 3.1. Emulsion Preparation and Stability

To prepare NEO-loaded microcapsules, the sodium alginate solution concentration was chosen first. Some pilot research has been done about sodium alginate amount in emulsion with nutmeg essential oil, and it was determined that 4% solution of sodium alginate is the best media for dilution with distilled water and other added materials. For this research, sodium alginate solution was prepared by not stirring or heating; instead, the powder of sodium alginate was spread on the water surface and left there for 24 h at room temperature, and the glass was covered to avoid environmental factors. This way the homogenous solution was prepared without air bubbles or some agglomerates of sodium alginate. Using the sodium alginate solution, we prepared five different groups of emulsions with the nutmeg essential oil; the compositions of all emulsions are presented in Table 1. In the first group of emulsions, we checked the possibility of polysorbate to be used as the emulsifier; in the second group, we determined the morphology and stability of emulsion made of equal parts of the nutmeg essential oil and polysorbate (1:1 ratio); in the third group, we chose fixed amounts of polysorbate and nutmeg essential oil and changed the amount of sodium alginate; in the fourth group, we added magnesium aluminometasilicate to the emulsion T2; and in the fifth group, we used a new emulsifier, sucrose esters (Sisterna SP70). 

**Figure 2:**
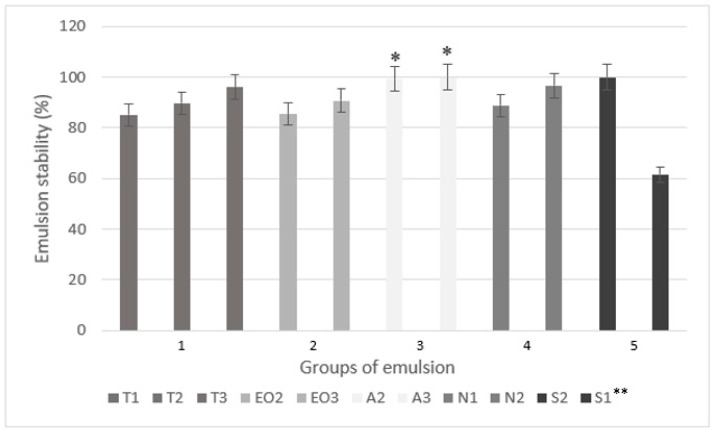
The emulsion stability (centrifugation index) using different amounts of excipients. * *p* < 0.05 vs. emulsion with a smaller amount of sodium alginate (T2). ** The code is given in Table 1.

In another study on the microencapsulation of essential oil by extrusion method, only sodium alginate (4%) and 1% of dye solution with rosemary essential oil were used [40]. In that research, the stability (CI) of emulsion was not determined, only parameters of microcapsules were investigated. The soy protein was used as an emulsifier and thyme oil loaded microcapsules (beads) had optimal parameters when using 1–2.5% of sodium alginate and 0–1.5% of soy protein [17] or 1–2% of sodium alginate and 0.5–1.5% of Tween 40 [18]. In our study, sodium alginate concentration in the emulsions varied from 1.23% (T1) to 3.51% (A3). The emulsion stability is presented in Figure 2. 

In the extrusion method for microencapsulation, sodium alginate is the most used shell material at the moment, although chitosan may also be used [44]. The results of the earliest studies mentioned in the review of microencapsulation technology stated that it is possible to use carbohydrates (starch, maltodextrins, corn syrup solids, dextran, cyclodextrins) as the shell material in the extrusion method. Meanwhile, sodium alginate’s properties have been used in chewing gum but not in extrusion [45]. New studies have shown that sodium alginate could be used in extrusion and could formulate microcapsules with essential oil as the core material [26,28].

In this research, the sucrose esters were used as the emulsifier to increase essential oil emulsion stability and to replace polysorbate. In addition, magnesium aluminometasilicate, which can absorb volatile compounds (in the previous study it was determined that this material absorbs and increases the yield of nutmeg essential oil and some volatile compounds’ amounts [42]), was used for the first time. 

The following emulsions, T3, A2, A3, N2, and S2 had the highest stability (CI was higher than 96%, Figure 2). An increased emulsifier amount (polysorbate and sucrose esters) increased the stability of nutmeg essential oil emulsions, as well as the sodium alginate did. Compared to the emulsion without an excipient (T2), 0.2 g of magnesium aluminometasilicate (N1) decreased emulsion’s stability but the CI was higher when using 0.4 g of excipient (N2); unfortunately, the results were not significant. The lowest CI was in emulsion with the lowest amount of sucrose esters (S1) (only 61.53%) but this group of emulsions (5) also had the highest amount of the nutmeg essential oil. In the EO3, which had the same amount of essential oil with equal amount of polysorbate, CI was 90.68%. The results showed that 0.5 g of sucrose esters can ensure stability in emulsion with 1.5 g of essential oil. When comparing sodium alginate’s influence on CI, the A2 and A3 emulsions were significantly more stable than the T2 (the emulsion with the lowest amount of sodium alginate but the same amount of other materials, Figure 2).

The primary emulsions for microcapsules were prepared similarly in all studies [23,26,33], including this study. Yet in this research, three different methods were used for group 5 emulsions (Figure 1) to determine which method is the most effective and results in the highest CI. The results of the remaining two methods are not reflected in Figure 2 (only method B results, Figure 1), as their CI was lower than 45%. The best method to prepare emulsion with Sisterna SP70 is when the nutmeg essential oil is added last.

In oil in water (o/w) emulsions, the amount of sucrose esters varies from 0 to 0.35%. The most stable emulsion was made using milk fat, skim milk powder, hydrocolloids, and sucrose. It was prepared by using 0.1% of sucrose esters, although guar (0.1%), xanthan (0.05%), and carrageenans (0.05%) were also used [23]. Approximately, 1% sucrose esters were used as an emulsifier in the making of other o/w emulsions (5% and 30% of flavor compounds were used), and the influence of emulsion viscosity on volatile compounds was determined [46]. Our study showed that 1.18% sucrose esters in the nutmeg essential oil emulsion (S1) emulsifies the oil but the emulsion is unstable, which cannot be said about 2.9% sucrose esters in the S2 emulsion. 

### 3.2. The Study of Emulsion and Microcapsule Morphology

**Figure 3:**
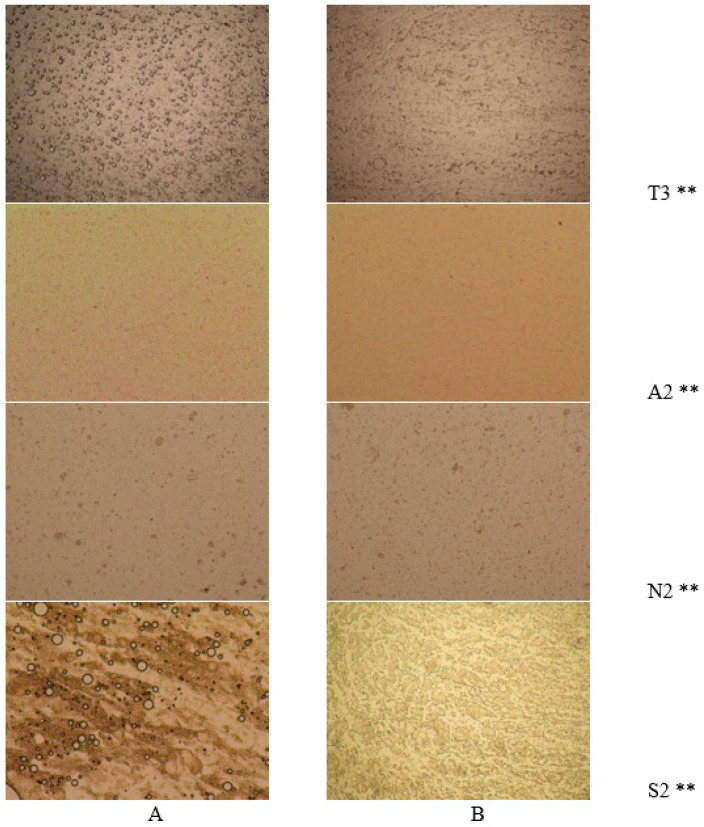
Emulsion morphology study in NEO-loaded emulsions using different materials. (**A**) The emulsions before homogenization and (**B**) after homogenization (magnification 10×) ** The code is given in Table 1.

The structures of emulsions with different excipients are not similar (Figure 3). Using Nikon microscope (zoom 10×), the emulsion morphology and drop areas were measured. The most homogenous emulsions before homogenization were the A2 and N2 because drop areas were the smallest (5.01 ± 1.42 nm and 8.21 ± 7.10 nm) and the view of the surface was smooth.

After homogenization, the surface of the S2 emulsion changed the most (Figure 3B and S2), and the drops acquired irregular shapes. The drop area changed the most in the T3 emulsion; the diameter after homogenization significantly decreased (from 14.75 ± 5.79 nm to 8.09 ± 5.09 nm). In other studies, the emulsion drops with sodium alginate decreased when the shell material amount was increased from 5 g/L to 45 g/L (from 100–400 µm to 10–50 µm, respectively) [36]. Our results were similar to the results of this study: when 0.2 g sodium alginate in sample T3 increased to 0.6 g in A3, the drop size also decreased (from 14.75 ± 5.79 nm to 4.00 ± 1.47 nm). Sodium alginate has emulsifying properties, though they are poor [17].

Chitosan has a significant influence on homogeneity of emulsion; compared with alginate emulsion, the drops are much larger (homogenization speed 10,000 rpm, 1.997 µm and 2.565 µm) [41]. In this research we did not use different shell materials, so the influence of different emulsifiers on the emulsion structure was compared. The emulsion with sucrose esters had a heterogeneous appearance (Figure 3A and S2); after homogenization (Figure 3B and S2), the view cardinally changed, the structure of drops changed from round to irregular forms (long and elongated, similar to mesh). Using polysorbate 80, the structure of drops after homogenization (Figure 3B, T3 and A2) did not change, the magnesium aluminometasilicate and a higher amount of sodium alginate also did not have any influence on the form of drops.

**Figure 4:**
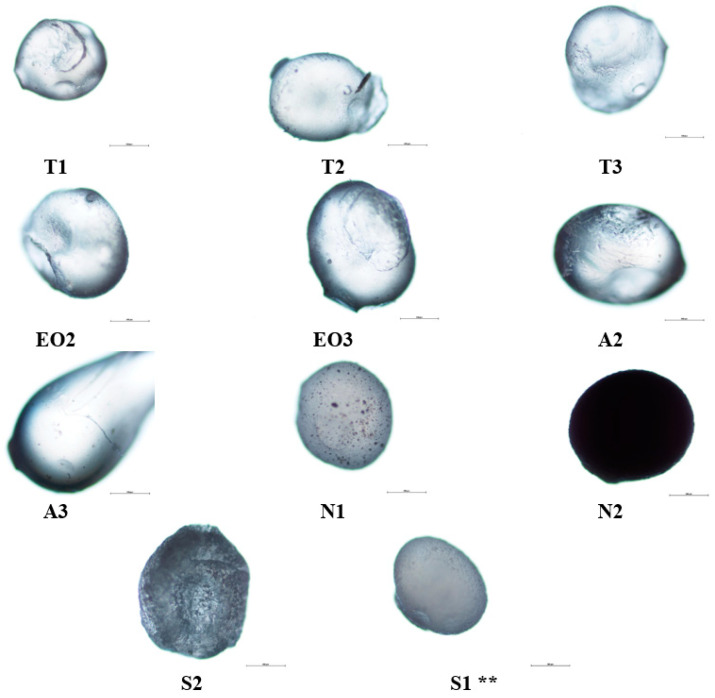
NEO-loaded microcapsules’ (dry) surface structural changes using different materials, 2% calcium chloride solution was used. ** The code of microcapsules is given in Table 1. The scale bar by each microcapsule is 500 µm.

Studying microcapsules’ surface and structure (Figure 4), the influence of sodium alginate amount was determined: it increased the size of the capsules and the A3 capsules were deformed (had a tail) compared with the T1 and A2 because the A3 emulsion was the thickest. In N1 NEO-loaded microcapsules, magnesium aluminometasilicate dots were visible; when the excipient amount was increased, the capsules became opaque, the light could not pass through the microcapsule and the view was almost black (N2).

Sucrose esters also had an impact on nutmeg essential oil microcapsules: a large amount of emulsifier (S2) changed the capsule’s surface, it was rough and uneven, and the capsule was not as translucent as NEO-loaded microcapsules with polysorbate 80. Magnesium aluminometasilicate had a lower influence on the surface than sucrose esters, it made the microcapsule surface rugged (formed deep wrinkles).

Authors of a study about sodium alginate and alginate/chitosan microcapsules [18] present the differences in microcapsule structure. The alginate microspheres have a smooth surface, and the alginate/chitosan have a noticeably irregular surface.

**Figure 5:**
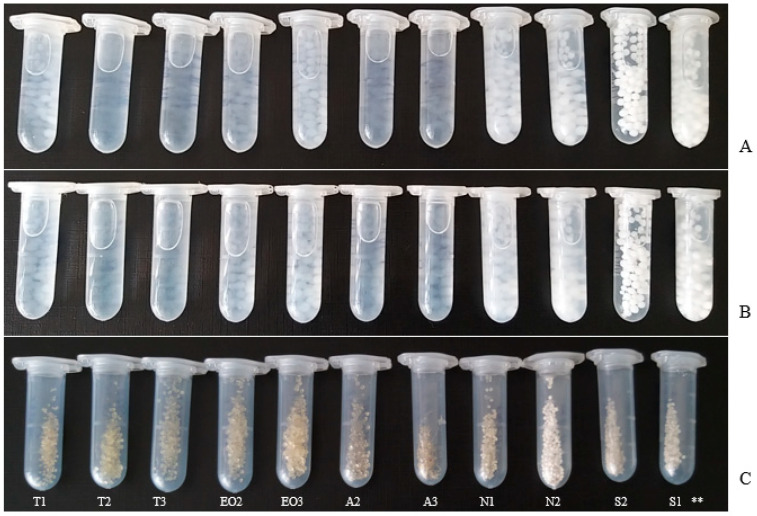
NEO-loaded microcapsules. Wet capsules in the crosslinker solution: (**A**) 2% solution; (**B**) 5%; (**C**) dry microcapsules (2% crosslinker solution). ** The code of microcapsules is given in Table 1.

The NEO-loaded freshly made calcium alginate microcapsules were photographed in 2% and 5% crosslinker solution (Figure 5A,B), and after one day, dry capsules were as well (Figure 5C). Magnesium aluminometasilicate and sucrose esters made microcapsules opaque (wet NEO-loaded microcapsules). When capsules were dry, the capsules with polysorbate 80 were yellow or light brown color, with sucrose esters and magnesium aluminometasilicate that were opaque with a white undertone.

### 3.3. Physical Parameters of NEO-Loaded Calcium Alginate Microcapsules 

Studying the parameters of NEO-loaded microcapsules (Table 2), the influence of crosslinker concentration on particle physical parameters was analyzed. It was determined that in all capsules, the nutmeg essential oil encapsulation efficiency was higher when 2% of calcium chloride was used, although in NEO-loaded microcapsules with magnesium aluminometasilicate (group 4), the results were opposite: the higher concentration of crosslinker ensured higher encapsulation efficiency. Yet magnesium aluminometasilicate alginate microcapsules had a lower EE because this excipient absorbs volatile compounds and they cannot dissolute in ethanol solution, so it locks nutmeg essential oil in microcapsules and the nutmeg oil is not released. This can be assumed because, when comparing the EE of the N1 and T1 microcapsules where the only difference is the content of magnesium aluminometasilicate, their encapsulation efficiencies were similar, while the increased excipient content decreased the release of essential oil from 45.55 ± 3.45% to 39.65 ± 1.51%. The highest EE was determined in microcapsules with sucrose esters and in the EO3 where the highest quantity of nutmeg essential oil was used. When comparing the EE of microcapsules from sodium alginate group (group 4, the T2, A2, and A3 microcapsules), it was noticed that the increased alginate amount decreased the encapsulation efficiency (Table 2, T2, A2, and A3). It could also formulate harder microcapsule shells and the nutmeg essential oil was not released from the particles (comparison between the groups only, the crosslinker concentration is not taken into account). The A3 sample with 5% of crosslinker was different and had a higher EE than the A2, but not significantly. From these results the correlation between polysorbate and nutmeg oil amount was determined (1:1, T1, EO2, EO3); the increased amount of these substances in emulsions increased the encapsulation of oil in microcapsules (from 41.89 ± 1.91% to 78.30 ± 3.44%). To compare with Yilmazktekin et al. [35], a higher quantity of essential oil but a smaller amount of sodium alginate determined the lower EE (2 g essential oil with 18 g sodium alginate, 98.4 ± 4.3%; 4 g with 16 g, 96.5 ± 5.5%).

To conclude the CI (Figure 2) and EE results (Table 2), the direct relationship between emulsion stability (CI%) and encapsulation efficiency (EE%) could not be established. In one case (group 2), the EE increased while increasing the stability of emulsion, but in other specimens (group 5) everything was the opposite. More physical studies would be needed to determine if the stability of the emulsion affects the encapsulation of the essential oil.

**Figure 6:**
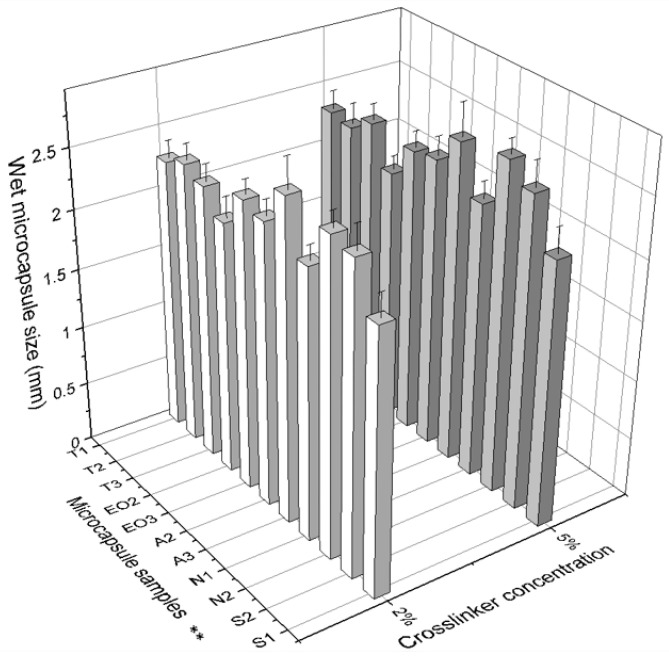
The effect of crosslinker concentration on the size of dry microcapsules. ** The code is given in Table 1, *n* = 30.

Microcapsule diameter (Table 2) depends on the amount of materials: in the first group (T) there was a direct dependency on polysorbate content: when the amount of Tween 80 was increased, the size of dry NEO-loaded microcapsules also increased. In addition, a lower concentration of CaCl_2_ solution resulted in smaller size capsules (Figure 6). No significant influence was found in either group when a standard deviation was calculated. We found that the amount of sodium alginate had more influence on the capsule size than other materials: it is the shell material and its amount that increases the capsule surface area. 

**Figure 7:**
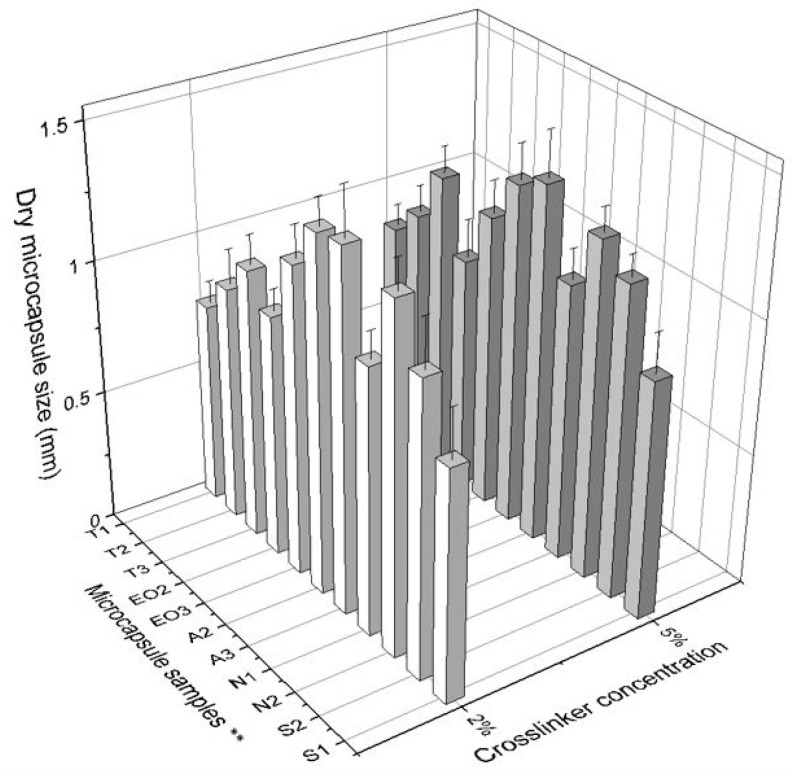
The effect of crosslinker concentration on the size of wet microcapsules. ** The code is given in Table 1, *n* = 30.

When comparing wet NEO-loaded calcium alginate microcapsules, it was determined that the largest capsules were obtained when 0.6 g of sodium alginate (A3) and 0.4 g of magnesium aluminometasilicate (N2) were used (Figure 7). Magnesium aluminometasilicate is a light powder that enlarges the skeleton of the capsule. The microcapsules’ diameter appertains to emulsion viscosity. In a study using only alginate, the diameter’s average was 2.37 mm, when the starch was added it was 2.48 mm, and using carrageenan and locust bean gum the diameter of microcapsules was the largest (3.72 mm) because the emulsion was the thickest [31]. In another study, the size of calcium alginate microcapsules (encapsulation of palm oil) was 1.83 mm [36]. Our emulsion with sodium alginate was the thickest but with magnesium aluminometasilicate the viscosity was medium. These results are due to the fact that magnesium aluminometasilicate is a very amorphous and lightweight powder, which forms a capsule skeleton when the capsule is made.

When 16 g of sodium alginate and 4 g of peppermint essential oil were used in yet another study, the peppermint microcapsule size was nearly 150 µm higher than that of 18 g of sodium alginate and 2 g of the essential oil (calcium chloride solution was 1.5%) [35]. 

There was no significant difference in the particle size when a different calcium chloride solution was used. The diameter of microcapsules (wet and dry) differed most when they were made using 0.6 g of sodium alginate (A3) (Table 2). This may have been due to the viscosity of emulsion. 

Another observation was that the size distribution of wet capsules was smaller compared to dry capsules. This scattering of sizes can be due to poorly encapsulated essential oil, which simply evaporated (Figure 6).

**Figure 8:**
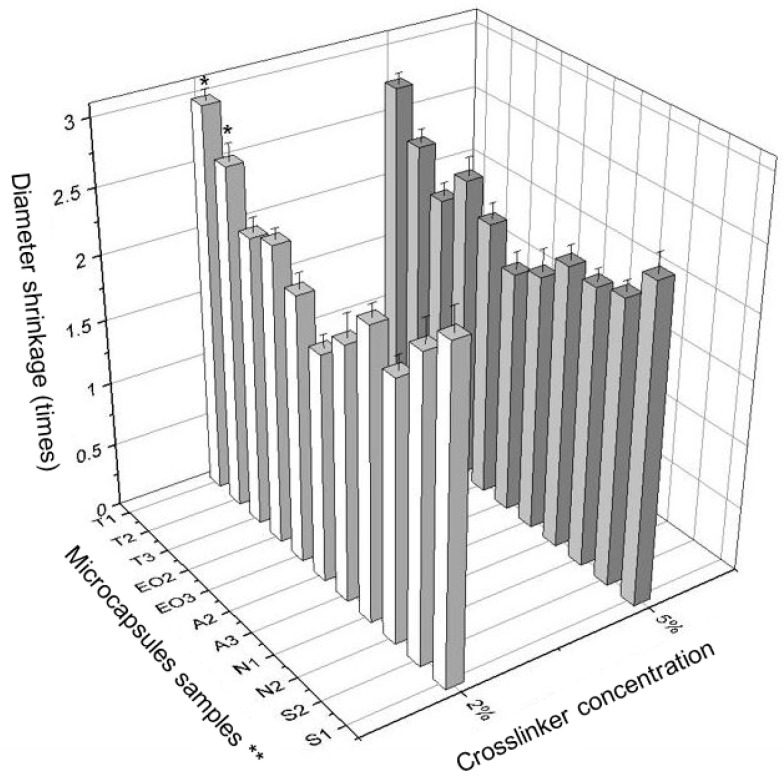
The difference in size (diameter shrinkage) between wet and dry microcapsules. * *p* < 0.05 vs. diameter of NEO-loaded microcapsules prepared in 5% crosslinker solution. ** The code is given in Table 1.

It was interesting to notice the diameter shrinkage between the wet and dry NEO-loaded calcium alginate capsules. Using this data, the differences were calculated (Figure 8). 

**Figure 9:**
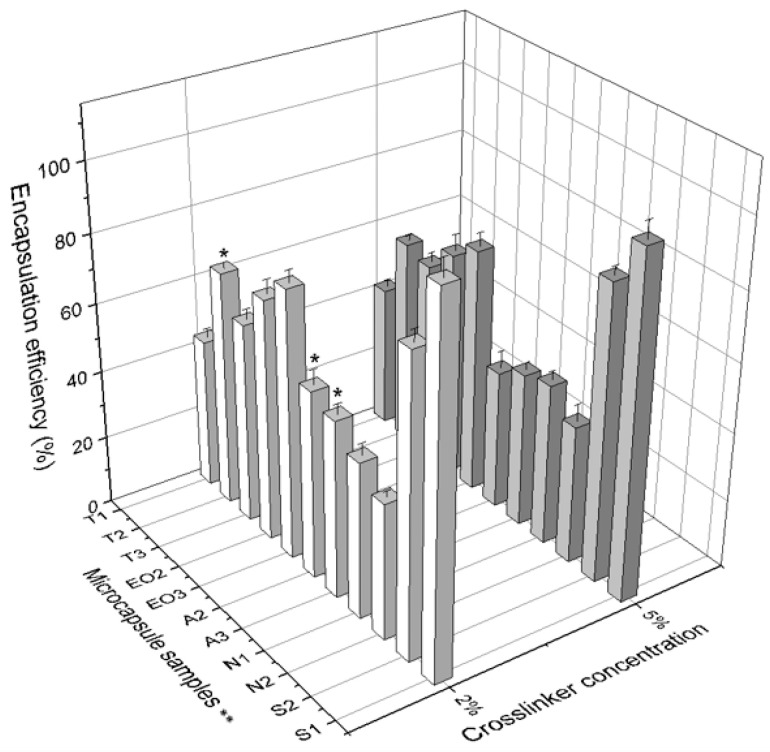
The comparison of encapsulation efficiency between 2% and 5% of crosslinker solution. * *p* < 0.05 vs. EE of NEO-loaded microcapsules prepared in 5% crosslinker solution. ** The code is given in Table 1, *n* = 3.

A significant difference between the effects of crosslinker concentrations was found. The difference between the wet and dry T1 and T2 microcapsules size (diameter shrinkage) was significant. The NEO-loaded microcapsules prepared using 2% of calcium chloride decreased less when drying than the capsules prepared with 5% of this solution (Figure 8). Using a lower concentration crosslinker solution, the essential oil was better encapsulated, the fresh NEO-loaded microcapsules were smaller, but the difference between wet and dry microcapsules was higher (in 6 of 11 samples, Figure 9). 

This study was supported by other results determined in earlier studies. Calcium alginate capsules with glucose oxidase were prepared with different crosslinker concentrations (1.3%, 2.6%, 4%, and 5.5%). The core material EE and microcapsule diameter were measured. The correlation between crosslinker solution and EE was not found, but when a higher concentration of calcium chloride was used, the diameter increased (sodium alginate 0.5%; the crosslinker solution concentration changed from 1.3% to 4.0%, the diameter was 6.4 ± 0.1, 7.4 ± 0.1, and 8.4 ± 0.1 mm). The same results were obtained with the increased sodium alginate’s concentration [39]. 

Figure 9 shows that the amount of sodium alginate had a larger influence on the EE when using 2% of calcium chloride compared with the influence on EE when 5% crosslinker solution was used (T2 68.79% vs. 60.59%; A2 54.82% vs. 40.77%; A3 51.79% vs. 45.27%). The crosslinker concentration had an influence on the EE of NEO-loaded microcapsules with different quantities of sodium alginate (shell material). We determined that 9 of 11 samples of NEO-loaded capsules prepared in 2% of calcium chloride solution had a higher essential oil encapsulation efficiency compared to 5% crosslinker solution (Figure 9).

The last physical parameter of microcapsules is firmness. It was measured by a texture analyzer and the force (g force) needed to break (rupture and crush) the NEO-loaded microcapsules was calculated (Table 2). All samples of microcapsules which were prepared using 5% crosslinker solution had higher firmness than the capsules prepared with the lower concentration of calcium chloride solution (Figure 9).

**Figure 10:**
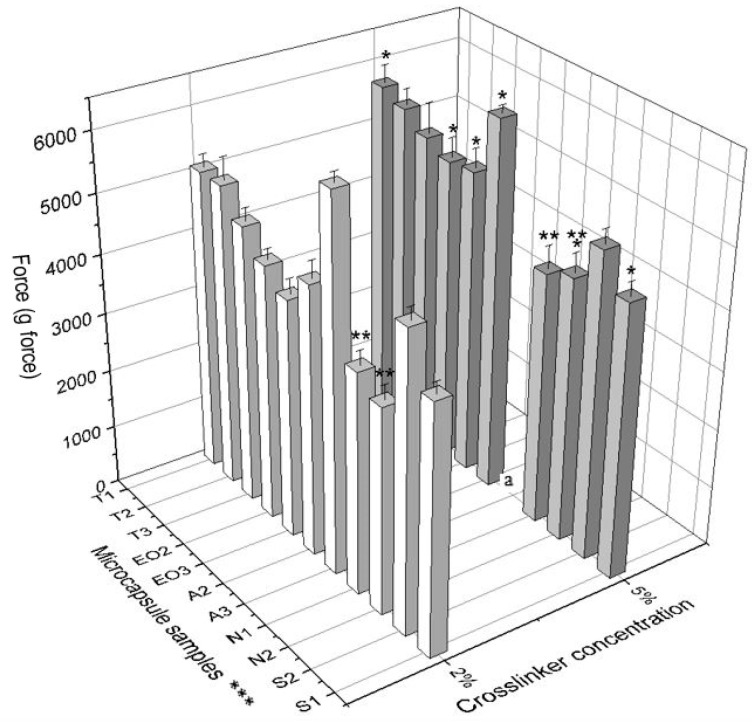
The firmness of NEO-loaded calcium alginate microcapsules. * *p* < 0.05 vs. force for crushing NEO-loaded microcapsules prepared by using 2% crosslinker solution. ** *p* < 0.05 vs. force for crushing NEO-loaded microcapsules prepared by using magnesium aluminometasilicate as excipient. A force for crushing A3 (5% calcium chloride solution) was not measured, it was higher than 6500 g force, the device’s maximum force is only 6500 g force. *** The code is given in Table 1, *n* = 3.

In the group 2 (EO2 and EO3), both samples were prepared using 5% calcium chloride and they had a significantly higher firmness than samples prepared using 2% crosslinker solution. An interesting observation is that magnesium aluminometasilicate decreased the firmness of microcapsules. To compare the forces for crushing T2 and N1 with N2, we saw that the excipient significantly decreased NEO-loaded microcapsule firmness (2% crosslinker solution: 5128.76 g force vs. 3732.38 g force vs. 3476.68 g force; 5% crosslinker solution: 5547.78 g force vs. 4261.80 g force vs. 4457.20 g force) (Figure 10). Consequently, the crosslinker concentration had an influence on NEO-loaded microcapsule firmness and some excipients had as well.

The firmness was not determined in other investigations of microcapsules prepared by extrusion. 

### 3.4. Swelling Characteristic of NEO-Loaded Microcapsules

The swelling was performed on all sample microcapsules prepared in 5% crosslinker solution. The swelling study consisted of several parts: SGF and SIF enzyme-free swelling; swelling using pepsin and pancreatin in the same media; capsules swelling in SGF medium for an hour and then being transferred to SIF (gastro-intestinal media). 

**Figure 11:**
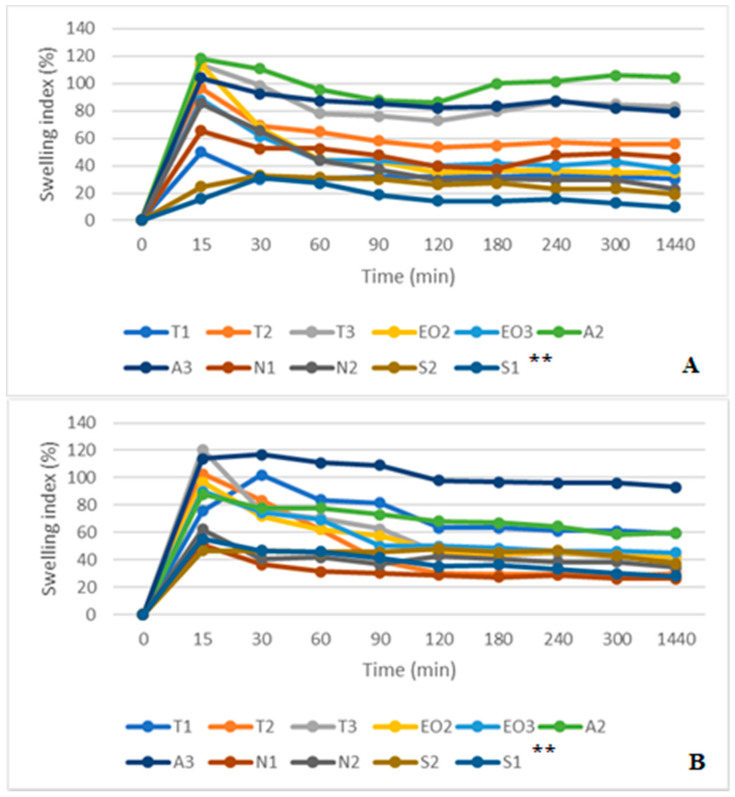
(**A**) NEO-loaded calcium alginate microcapsules swelling in simulated gastric juice (SGF) medium. (**B**) NEO-loaded calcium alginate microcapsules swelling in SGF+ pepsin medium. ** The code is given in Table 1, *n* = 3.

Comparing SGF and SGF+ pepsin swelling results (Figure 11A,B), all sample capsules reached maximum swelling after 15 min in simulated gastric medium. Then the SGF was used, the highest swelling was in A2 and T3 NEO-loaded capsules (after 15 min it was 118.18% and 113.56%, respectively). In this medium, S1 and S2 microcapsules had the smallest swelling index (after 15 min it was 15.71% and 24.66%, after one day (1440 min) it was 10.00% and 19.18%, respectively). Microcapsules with sucrose esters as an emulsifier had a lower swelling index in SGF medium. 

**Figure 12:**
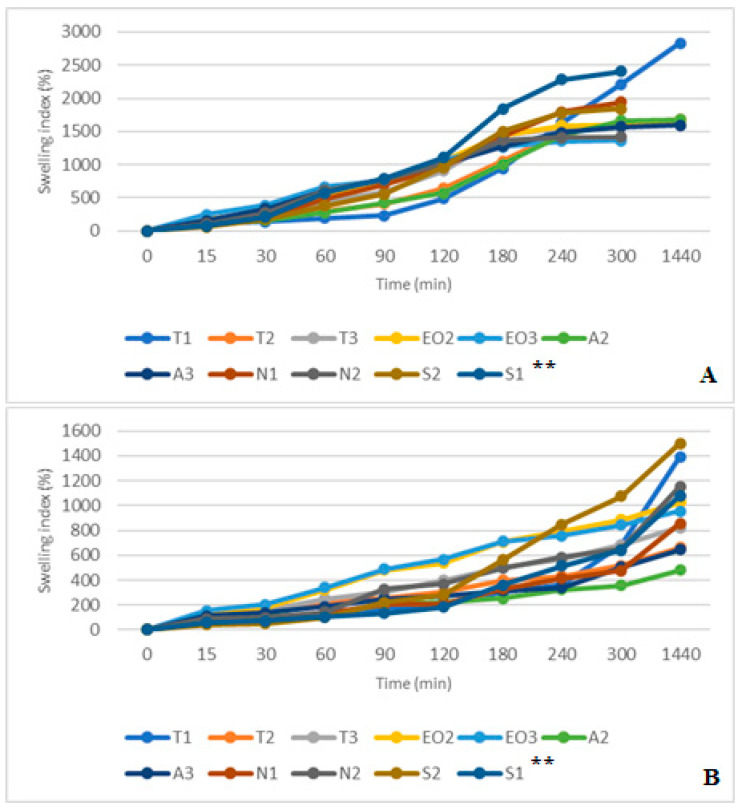
(**A**) NEO-loaded calcium alginate microcapsules swelling in simulated intestinal juice (SIF) media. (**B**) NEO-loaded calcium alginate microcapsules swelling in SIF+ pancreatin media. ** The code is given in Table 1, *n* = 3.

The results in SGF+ pepsin medium were not the same as in SGF medium because T1 and A3 NEO-loaded microcapsules reached maximum swelling index in 30 min (from 75.51% and 114.00% at 15 min to 102.04% and 117.00% at 30 min, respectively). The highest swelling index after a day was in A2 (93.00%) and A3 (59.76%) microcapsules. Consequently, these results were the same as in SGF medium (the A3 sample was in the third place, its swelling index was a little bit less than that of the T3, Figure 11). Pepsin did not significantly change the swelling index of NEO-loaded capsules, but results in SIF and SIF-pancreatin media were different (Figure 12A,B). First of all, the swelling index was higher in SIF media. After 300 min of study, capsules of a few samples turned into a gruel (T3, EO3, N1, N2, S2, and S1), their SI in 1440 min was not measured (Figure 12A). This was not the case with capsules in SIF+ pancreatin medium, all of which retained their structure after 1440 min and were easily drained without crushing (Figure 12B). 

The pancreatin powder had an influence on the swelling index. It prevented capsules from unlimited swelling. After one day, all NEO-loaded microcapsules in both SIF media had a higher SI than in SGF medium. In SIF medium, T1 microcapsules were the most swollen (2835.82%), while in SIF+ pancreatin medium the swelling was less than half of that (1393.33%). Comparing results at 300 min we saw that the S2 had a higher SI (SIF+ pancreatin, 1074.14%); this index in SIF medium was achieved after 180 min. A2 and A3 capsules had the lowest swelling index (478.89% and 645.16%, Figure 12B). Sodium alginate as a shell material protected the capsules from excessive swelling.

**Figure 13:**
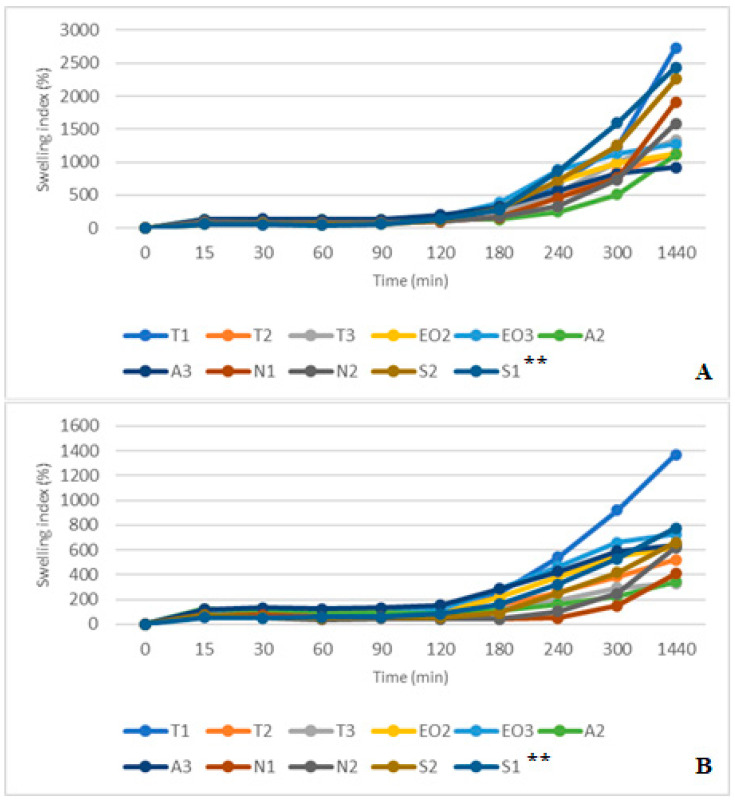
(**A**) NEO-loaded calcium alginate microcapsules swelling in SGF and SIF media. (**B**) NEO-loaded calcium alginate microcapsules swelling in SGF+ pepsin and SIF+ pancreatin media. ** The code is given in Table 1, *n* = 3.

By combining the study results of two media (gastric and intestinal), it was found that gastric acid (with and without pepsin) prolongs swelling of the capsules in the intestinal medium. They swelled less rapidly; it took about an hour to reach normal swelling compared to the swelling time in a separate SIF medium (Figure 13A,B). The media change had less influence on T1 NEO-loaded microcapsules, T1 had a higher SI in both studies, and similar results were found in SIF and SIF-pancreatin media.

Unlike in SIF results, all capsules maintained their shape after 1440 min (Figure 13A). All capsules could be drained without crushing because their SI was lower. In this swelling study, we did not determine the amount of released nutmeg essential oil compounds. Studies are planned to be carried out in the future using several formulations of microcapsules containing the excipients described in this article and with new formulations, with other excipients.

Volic et al.’s [17] swelling study with alginate and soy protein microcapsules (thyme essential oil is encapsulated) established that in SGF medium microcapsules lose weight. When the investigation was continued after changing the medium to SIF, the capsule weight grew but only until a certain point. The same results were obtained by Azad et al. [34]: alginate/lecithin beads swelled in SGF and SIF media without enzymes. In SGF medium the beads reached maximum swelling in 40–60 min, then the SI decreased. Swelling in SIF medium results showed that microcapsules reached SI over 1000% in 80 min and after 90 min SI decreased. In this study, SI did not decrease in SIF media but increased all the time until it slowly stopped when microcapsules lost their shape. 

Dima et al. [47] confirmed the results of this research: alginate microcapsules swelled much better in intestinal medium (pH = 6.5–7), while in gastric juice (pH = 1–2.5) alginate microcapsules almost did not swell; although the chitosan microcapsules behaved oppositely. They swelled in SGF and did not swell in SIF.

## 4. Conclusions

In this study, two new materials, sucrose esters and magnesium aluminometasilicate, were used for encapsulation of nutmeg essential oil by extrusion. Both of these materials affected the nutmeg essential oil encapsulation efficiency, microcapsule swelling index, and other physical parameters compared to the influence of polysorbate 80 on NEO-loaded microcapsules.

New emulsifier Sisterna SP70, which was used in extrusion for the first time, could also ensure the stability of emulsion but its amount should not be lower than 3:1 (nutmeg essential oil:sucrose esters). When using this emulsifier, the nutmeg essential oil encapsulation was the highest in all samples (from 86.08% to 99.82% when crosslinker was 5%), so it is a good material to ensure NEO encapsulation and protection from environmental impact. The other excipient, magnesium aluminometasilicate, had a different effect on microcapsules: it absorbed the essential oil, which was then difficult to release from the microcapsules by ethanol solution and the encapsulation efficiency was lower in all the samples (39.65–45.55%, 2% of CaCl_2_ solution). The largest diameter of the microcapsules was when magnesium aluminometasilicate was used (wet capsules, 2.751 mm; dry capsules, 1.300 mm). However, the hardness was not affected by sucrose esters and magnesium aluminometasilicate. The capsule swelling test showed a very high capsule swelling index in the intestinal medium (higher than 1400% after 300 min), when pancreatin powder was added, the SI maximum range was 1074.14%. In further studies, it is appropriate to determine the release of the active compounds in simulation media.

## Figures and Tables

**Table 1:** The composition of emulsions from which the nutmeg essential oil (NEO)-loaded microcapsules were made.

Group	Code	Sodium Alginate (g)	Polysorbate 80 (g)	Nutmeg Essential Oil (g)	Distilled Water (mL)	Magnesium Aluminometasilicate (g)	Sucrose Esters (g)	Amount of Excipients ^a^ (g)
1	T1 ^b^	0.2	0.5	0.5	15	-	-	0.7
	T2 ^c^	0.2	1.0	0.5	15	-	-	1.2
	T3	0.2	1.5	0.5	15	-	-	1.7
2	EO2	0.2	1.0	1.0	15	-	-	1.2
	EO3	0.2	1.5	1.5	15	-	-	1.7
3	A2	0.4	1.0	0.5	15	-	-	1.4
	A3	0.6	1.0	0.5	15	-	-	1.4
4	N1	0.2	1.0	0.5	15	0.2	-	1.4
	N2	0.2	1.0	0.5	15	0.4	-	1.6
5	S2	0.2	-	1.5	15	-	0.5	0.7
	S1	0.2	-	1.5	15	-	0.2	0.4

^a^ Excipients: core material–sodium alginate; emulsifier–polysorbate 80, Sisterna SP70; magnesium aluminometasilicate. ^b^ T1 emulsion also is EO1 emulsion. ^c^ T2 emulsion also is A1 emulsion.

**Table 2:** Parameters of NEO-loaded microcapsules (crosslinker CaCl_2_ concentration; diameter of dry and just made (wet) microcapsules (mm); force required to crush wet capsules (g force); efficiency of encapsulation (%)).

Code **	CaCl_2_ Concentration (%)	Microcapsules Diameter (mm)		Force for Crushing (g Force)	EE (%)
		Wet	Dry		
T1 ^a^	5	2.317 ± 0.15	0.842 ± 0.08	5702.66 ± 295	41.89 ± 1.91
	2	2.280 ± 0.14	0.763 ± 0.08	5116.70 ± 209	43.88 ± 2.46
T2 ^b^	5	2.286 ± 0.17	0.951 ± 0.10	5547.78 ± 260	60.59 ± 1.35
	2	2.368 ± 0.13	0.892 ± 0.13	5128.76 ± 379	68.79 ± 1.54
T3	5	2.419 ± 0.13	1.152 ± 0.10	5290.20 ± 497	58.35 ± 2.74
	2	2.300 ± 0.14	1.024 ± 0.11	4711.92 ± 212	58.65 ± 2.92
EO2	5	2.110 ± 0.11	0.893 ± 0.14	5113.04 ± 322	66.12 ± 4.71
	2	2.120 ± 0.17	0.917 ± 0.08	4333.46 ± 180	70.53 ± 4.10
EO3	5	2.395 ± 0.13	1.115 ± 0.12	5173.51 ± 309	71.88 ± 3.75
	2	2.414 ± 0.12	1.166 ± 0.12	4044.36 ± 250	78.30 ± 3.44
A2	5	2.433 ± 0.15	1.290 ± 0.13	6252.33 ± 123	40.77 ± 4.89
	2	2.360 ± 0.13	1.336 ± 0.10	4554.90 ± 286	54.82 ± 4.34
A3	5	2.678 ± 0.27	1.348 ± 0.17	>6500	45.27 ± 1.88
	2	2.668 ± 0.25	1.353 ± 0.18	6245.63 ± 170	51.79 ± 2.87
N1	5	2.300 ± 0.14	1.049 ± 0.11	4261.80 ± 370	47.32 ± 2.52
	2	2.254 ± 0.12	1.004 ± 0.10	3732.38 ± 237	45.55 ± 3.45
N2	5	2.751 ± 0.12	1.272 ± 0.09	4457.20 ± 314	40.47 ± 4.81
	2	2.611 ± 0.13	1.300 ± 0.11	3476.68 ± 241	39.65 ± 1.51
S2	5	2.603 ± 0.21	1.177 ± 0.08	5160.73 ± 232	86.08 ± 2.41
	2	2.562 ± 0.20	1.101 ± 0.18	4989.43 ± 200	86.38 ± 3.31
S1	5	2.214 ± 0.21	0.900 ± 0.15	4610.1 ± 226	99.82 ± 4.94
	2	2.196 ± 0.19	0.867 ± 0.18	4174.12 ± 186	106.92 ± 4.90

EE = encapsulation efficiency. ^a^ T1 microcapsules also are EO1 NEO-loaded microcapsules. ^b^ T2 microcapsules also are A1 NEO-loaded microcapsules. ** The code is given in Table 1.
